# Supplementary material for: Spatial modeling of prostate cancer metabolic gene expression reveals extensive heterogeneity and selective vulnerabilities
Source: Sci Rep. 2020 Feb 26;10:3490. doi: 10.1038/s41598-020-60384-w (PMC7044328; doi:10.1038/s41598-020-60384-w)
Supplement: Supplementary file 1 — Supplementary Information. [file 41598_2020_60384_MOESM1_ESM.pdf]

Title: Spatial modeling of prostate cancer metabolic gene expression reveals extensive heterogeneity and selective vulnerabilities

Yuliang Wang[1,2*], Shuyi Ma[3], Walter L. Ruzzo[2,4,5]

[1]Institute for Stem Cell and Regenerative Medicine, University of Washington, Seattle, WA 98109, USA
[2]Paul G. Allen School of Computer Science & Engineering, University of Washington, Seattle, WA 98195, USA
[3]Center for Global Infectious Disease Research, Seattle Children's Research Institute, Seattle WA, USA
[4]Department of Genome Sciences, University of Washington School of Medicine, Seattle, WA, 98195, USA
[5]Fred Hutchinson Cancer Research Center, Seattle, WA, 98102, USA

*Correspondence should be addressed to Y.W. (email: yuliangw@cs.washington.edu)

Supplemental figures:

Figure S1

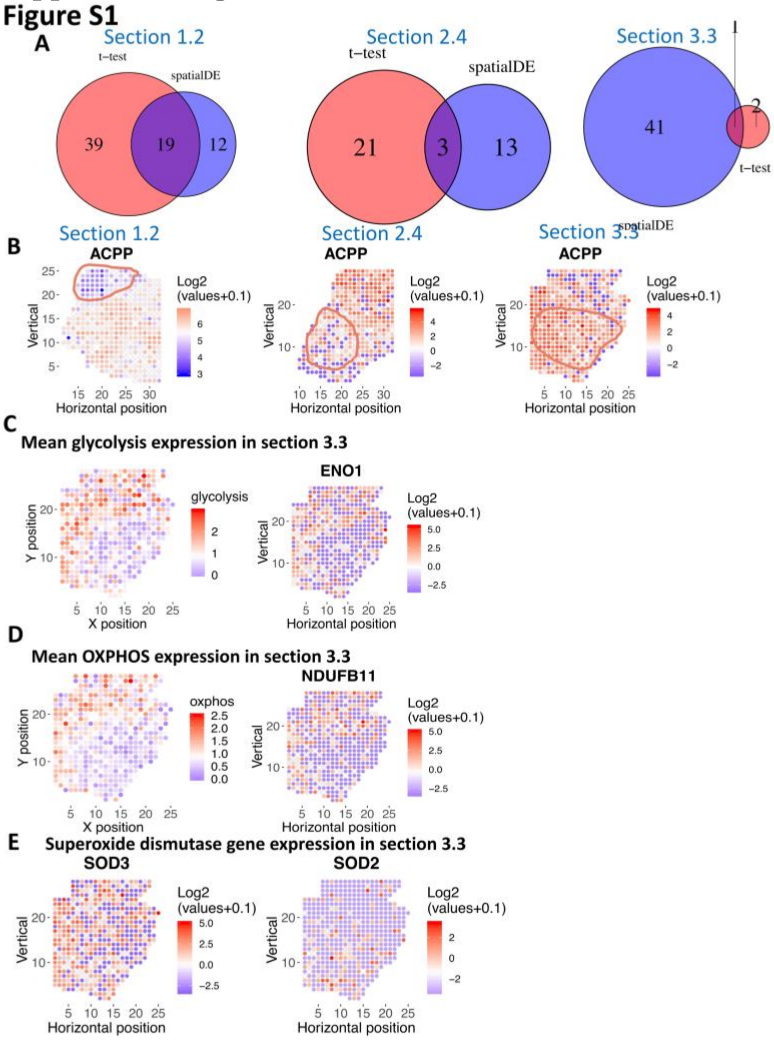

Figure S1.
A. Overlap of genes identified as spatially variable by spatial DE vs. t-test. The overlap is bigger when tumor region is well-defined and clustered together.
B. Log2 expression level of the ACPP (Acid Phosphatase, Prostate) gene, a known prostate cancer marker gene. Red color denotes higher expression; blue denotes lower expression. The ACPP gene is spatially variable across all three tissue sections.
C. Mean expression level of all spatially variable glycolysis genes (left) and an example, enolase 1 (ENO1) in section 3.3
D. Mean expression level of all spatially variable oxidative phosphorylation genes (left) and an example, NDUFB11 in section 3.3
E. Extracellular (SOD3) and mitochondrial (SOD2) superoxide dismutase show spatially distinct expression profiles. SOD2 has the opposite spatial distribution as OXPHOS (S1D).

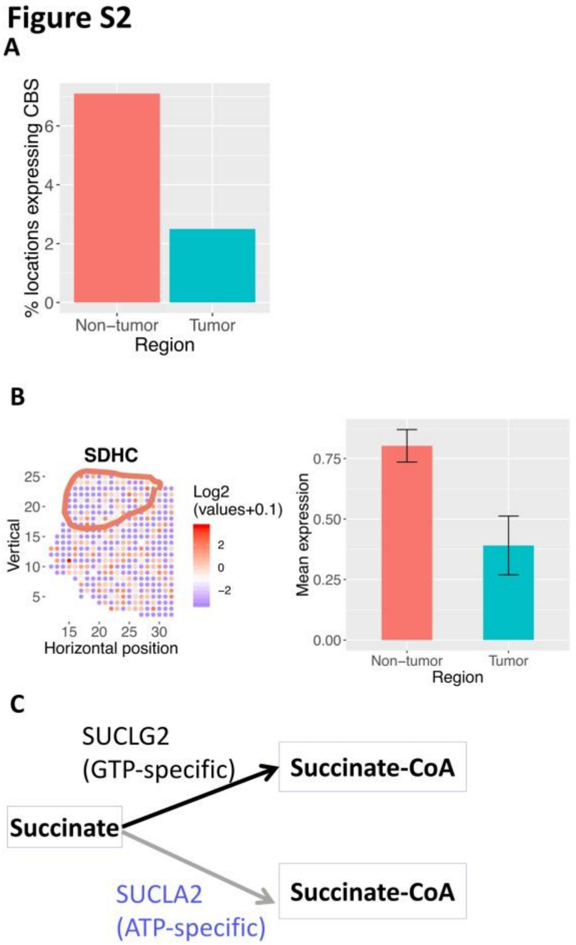

Figure S2.
A. Bar plot of percentage of locations within tumor and non-tumor regions that express CBS.
B. Succinate dehydrogenase is depleted in the tumor region of tissue section 1.2. Tumor region is circled. Left: log2 expression of SDHC across the tissue section. Red means higher expression; blue/white means low or no expression. Right: Mean expression of SDHC in non-tumor and tumor region. Error bar represents standard error of the mean.
C. Model predicted that in tissue section 2.4, SUCLG2 (GTP-specific succinyl-CoA synthetase) is lethal because the alternative route to produce succinate-CoA via SUCLA2 (ATP-specific

succinyl-CoA synthetase) is absent in the malignant region. Each rectangle represents a metabolite. Each arrow represents a reaction or transport (black arrow: reaction is present in the tumor; gray arrow: reaction is absent from the tumor). The name of each reaction is labeled above the corresponding arrow.

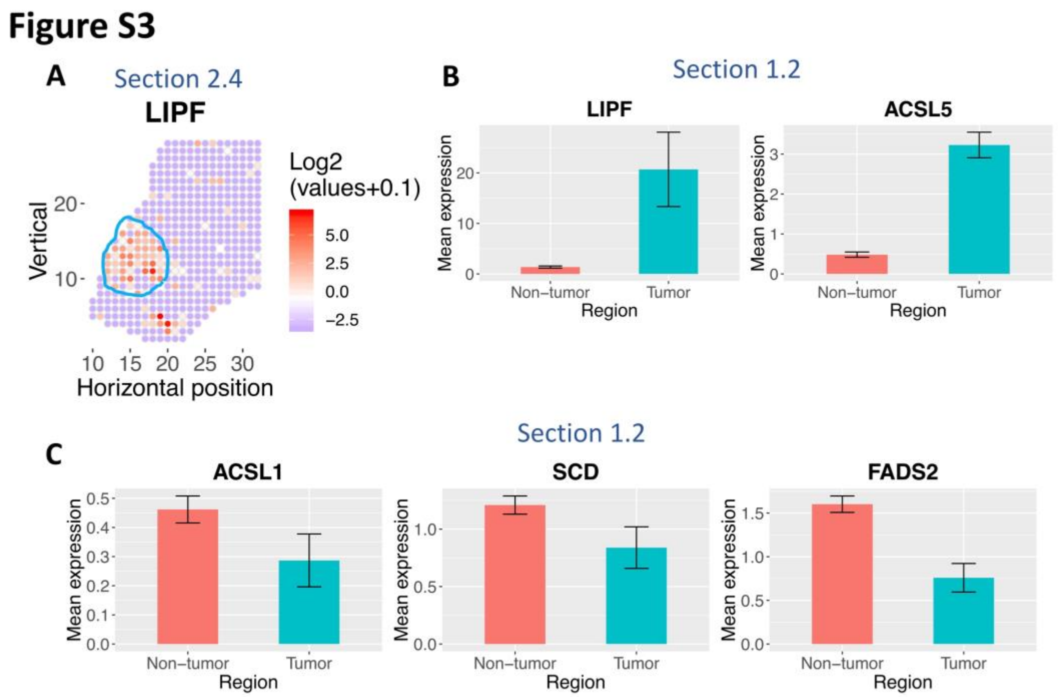

Figure S3.

A. Lipase F is also enriched in the tumor region in tissue section 2.4. Red color denotes higher expression; blue denotes lower expression. Tumor region is circled by blue lines.

B. Bar plot of mean expression level of lipolysis gene LIPF and fatty acid synthesis gene ACSL5 in section 1.2. . Error bar represents standard error of the mean.

C. Bar plot of mean expression level of fatty acid oxidation gene ACSL1, and fatty acid desaturation gene SCD and FADS2 in section 1.2.

Figure S4

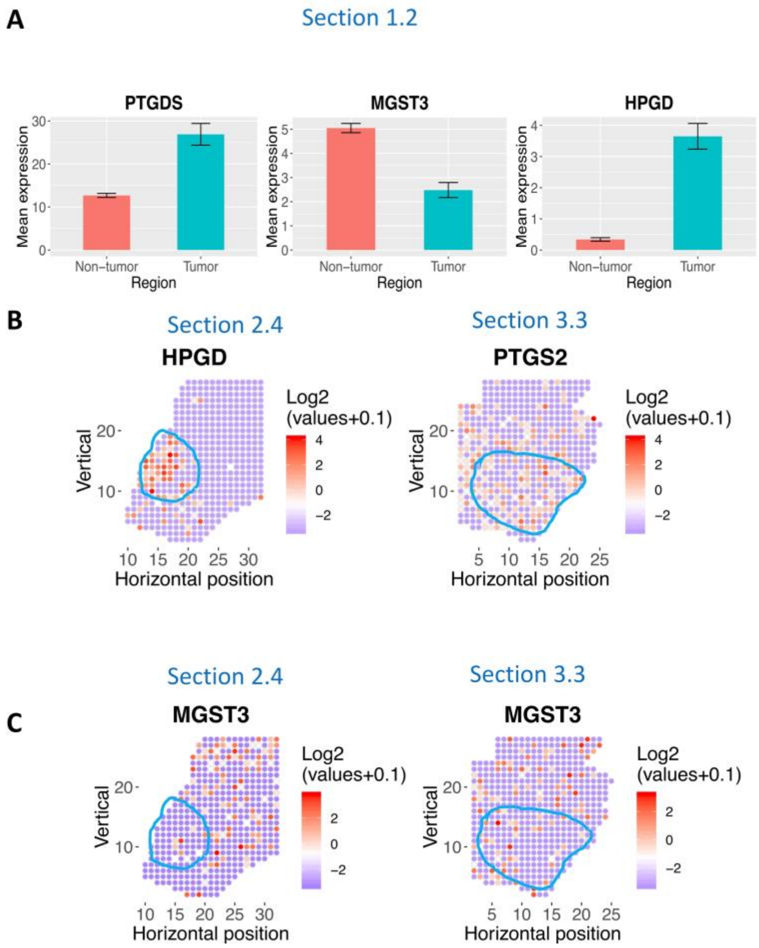

Figure S4.

Additional genes in arachidonic acid metabolism are spatially variable in prostate cancer

A. Bar plot of mean expression level of arachidonic acid metabolism genes in section 1.2. MGST3 is depleted in tumor region while PTGS and HPGD are enriched. Error bar represents standard error of the mean.

B. HPGD is enriched in the tumor region in tissue section 2.4; PTGS2 (i.e. COX-2), the first step in prostaglandin synthesis, is enriched in tumor region in section 3.3. Red color denotes higher expression; blue denotes lower expression. Tumor regions are highlighted in blue.

C. MGST3 is spatially variable and depleted in tumor regions in section 2.4 and 3.3 as well. Red color denotes higher expression; blue denotes lower expression. Tumor regions are highlighted in blue.
